# 

**Published:** 1890-08

**Authors:** 


### CONTRIBUTORS TO VOLUME XXX.

| | |
|---|---|
| Benedict, A. L., M. D. | Buffalo |
| Bennett, Alice, M. D. | Norristown, Pa. |
| Bergtold, William H., M. D. | Buffalo |
| Buswell, Henry C., M. D. | Buffalo |
| Dagenais, Alphonse, M. D. | Buffalo |
| Dorr, Samuel G., M. D. | Buffalo |
| Evans, Charles Seth, M. D. | Cincinnati |
| Falk, Jacob M., M. D. | Buffalo |
| Goldberg, Jacob, M. D. | Buffalo |
| Grove, B. H., M. D. | Buffalo |
| Hinkel, F. Whitehll, M. D. | Buffalo |
| Hoffman, Joseph, M. D. | Philadelphia |
| Hubbell, Alvin A., M. D. | Buffalo |
| Jackson, Edward, M. D. | Philadelphia |
| Krauss, William C., M. D. | Buffalo |
| Lothrop, Thomas, M. D. | Buffalo |
| Manley, Thomas H., M. D. | New York |
| Matteson, J. B., M. D. | Brooklyn |
| Miller, John A., Ph. D. | Buffalo |
| Mynter, Herman, M. D. | Buffalo |
| Park, Roswell, M. D. | Buffalo |
| Parmenter, John, M. D. | Buffalo |
| Penrose, Charles B., M. D. | Philadelphia |
| Potter, Frank Hamilton, M. D. | Buffalo |
| Potter, William Warren, M. D. | Buffalo |
| Price, Joseph, M. D. | Philadelphia |
| Putnam, James Wright, M. D. | Buffalo |
| Reed, Charles A. L., M. D. | Cincinnati |
| Rochester, DeLancey, M. D. | Buffalo |
| Roe, John O., M. D. | Rochester |
| Ross, James F. W., M. D. | Toronto |
| Sænger, Dr. Max | Leipzig |
| Shimwell, Benjamin T., M. D. | Philadelphia |
| Smith, Charles N., M. D. | Toledo |
| Smith, Eugene A., M. D. | Buffalo |
| Snow, Irving M., M. D. | Buffalo |
| Stanbro, Frederick, M. D. | Springville |
| Thomas, Charles Hermon, M. D. | Philadelphia |
| Thorner, Dr. E. | Berlin |
| Trowbridge, Grosvenor R. | Danville, Pa |
| Turnbull, Laurence, M. D. | Philadelphia |
| Wende, Ernest, M. D. | Buffalo |
| Wise, Peter. M., M. D. | Ogdensburgh |

### LIST OF ILLUSTRATIONS.

PAGE.

FRACTURE AND CONCUSSION OF TEMPORAL BONE AS A CAUSE OF DEAFNESS (L. A. TURNBULL).
Fig. 1.—Case of Fracture of Frontal and Parietal Bone, with Measurements after Recovery . . . . . . . . . . . . . . . . . . . . . . 3
PORTRAIT OF DR. ROBERT KOCH.
Facing . . . . . . . . . . . . . . . . . . . . . . . . . . . . . 299
OVARIAN AND LIGAMENTOUS CYSTS CO-EXISTING IN ONE PATIENT. (WILLIAM WARREN POTTER, M. D.)
Fig. 1.—Ovarian and Ligamentous Cysts . . . . . . . . . . . . . . . . 458
MUSCULAR ATROPHIES. A CLINICO - PATHOLOGICAL STUDY. (WILLIAM C. KRAUSS, M. D.)
Plate—Illustration of Muscular Atrophies, facing . . . . . . . . . . . . 513
DESCRIPTION OF PHOTOGRAPHIC PLATES OF AN EDEMATOUS ACARDIA. (JAMES F. W. ROSS, M. D.)
Plate—Edematous Acardia, facing . . . . . . . . . . . . . . . . . . 527
CONSTRUCTION AND ADAPTATION OF SPECTACLE FRAMES. (CHARLES HERMON THOMAS, M. D.)
Fig. 1.—Saddle-bridge—typical form (back view) . . . . . . . . . . . 593
Fig. 2.—Saddle-bridge, with horizontal arms; for prominent eyes and long lashes . . . . . . . . . . . . . . . . . . . . . . . . . . . 594
Fig. 3.—Saddle-bridge, with vertical arms for flattened nose; eyes high. . 595
Fig. 4.—Saddle-bridge; with clamps . . . . . . . . . . . . . . . . 596
Fig. 5.—Adapted Temple . . . . . . . . . . . . . . . . . . . . . 597
PORTRAIT OF DR. GEORGE N. BURWELL.
Facing . . . . . . . . . . . . . . . . . . . . . . . . . . . . . 641
OPERATIVE TREATMENT OF RECTO-VAGINAL FISTULA (DR. MAX SAENGER).
Fig. 1.—Denudation of the fistulous edges in the Vagina, with introduction of transverse sutures . . . . . . . . . . . . . . . . . . . . 643
Fig. 2.—A. Freshened edges. S. Suture. F. Fistula . . . . . . . . . 643
Fig. 3.—Triangular denudation. (After Schauta.). . . . . . . . . . . . 644
Fig. 4.—Closure of Fistula by transverse cresent-shaped lips. (After Fritsch). . . . . . . . . . . . . . . . . . . . . . . . . . . . 646
Fig. 5.—F. Fistula . . . . . . . . . . . . . . . . . . . . . . . . 646
Fig. 6.—A B and C D, sagitto-median incision through the Vagina. A E D and A F D, borders of the undermining of crescentic vaginal flaps. . . . . . . . . . . . . . . . . . . . . . . . . . . . . . 647
Fig. 7.—Vaginal flaps separated and turned back. Lauenstein's sutures applied as in Fig. 8 . . . . . . . . . . . . . . . . . . . . . . 647
Fig. 8.—1, 1. Buried suture of the rectal surface of fistula. 2, 2. Deep vaginal sutures. 3, 3. Superficial vaginal sutures. 4, 4. Rectal supportive sutures . . . . . . . . . . . . . . . . . . . . . . . . 648
Fig. 9.—Closure of the vaginal incision by deep sutures, seen from above. 649
Fig. 10.—Vaginal incision closed Introduction of rectal sutures . . . . 650
Fig. 11.—Simple perineal flap . . . . . . . . . . . . . . . . . . . 651
Fig. 12.—Fistula with only perineal fragment remaining . . . . . . . . 652
Fig. 13.—Perineo-vaginal bridge torn through . . . . . . . . . . . . . 652
Fig. 14.—Diagrammatic representation of operation . . . . . . . . . . 652
Fig. 15.—Anterior rectal wall closed by Lauenstein's deep sutures . . . . 653
Fig. 16.—Position of sutures after completed operation . . . . . . . . . 653

### Society Meetings.

---

The American Society of Microscopists will hold its next annual meeting in Detroit, Mich., Tuesday, Wednesday, Thursday, and Friday, August 12, 13, 14, and 15, 1890.

PROGRAMME OF SUBJECTS OF DISCUSSION:

Representation of the Society at the World's Fair, Chicago, 1893. Discussion to be opened by Ex-Gov. Jacob D. Cox, Cincinnati, Ohio; Micrometry, Prof. William A. Rogers, Waterville, Me.; Proposed Standing Committee on Medico Legal Microscopy, Prof. Marshall D. Ewell, Chicago, Ill.; Uniformity in Tube Lengths, Prof. Simon H. Gage, Ithaca, N. Y.; The Advisability of Adding more Members to the Publication Committee, Prof. D. S. Kellicott, Columbus, Ohio; Proposed New Constitution, Dr. William J. Lewis, Hartford, Conn.; The Advisability of Meeting at Same Time and Place of the American Association for the Advancement of Science, Prof. W. H. Seaman, Washington, D. C.; Advisability of Sending Copies of the Publications to some of the great Colleges and Libraries of the World, Dr. Lee H. Smith, Buffalo, N. Y.; Fees of Experts with the Microscope, C. M. Vorce, Esq., Cleveland, Ohio.

PAPERS.

The following titles of Papers to be read have been received up to the date of issue of this circular:

The Full Utilization of the Capacity of the Microscope, and Means of Obtianing the Same, Edward Bausch, Esq., Rochester, N. Y.; (1) The Structure of Protoplasm, (2) Microscope Objectives, Prof. T. J. Burrill, Ilinois State University, Champaign, Ill.; (1) Abnormal Forms in the Diatoms and Conclusions therefrom, (2) Review of Some of the Generic and Specific Distinctions in the Family *Coscinodisceæ*, Ex-Gov. Jacob D. Cox, Cincinnati, Ohio; (1) The Microscopic Identification of Hair, (2) The Effect of Curvature of the Cover Glass upon Micrometry, (3) Description of Scale, Manufactured by Marshall D. Ewell, in Pursuance of resolution of A. S. M., adopted in 1889, (4) A New Form of Stage Micrometer, (5) Some Experiments to Determine the Limit of Vision as Related to the Size of the Object Observed, (6) A Review of Some of the Medico-Legal Questions Involved in the Cronin Case, Prof. Marshall D. Ewell, Chicago, Ill.; Observations on the Blood in Health and Disease, Dr. Simon Flexner, Louisville, Ky.; (1) The Transition from Columnar to Stratified Epithelium, (2) Picric and Chromic Acid for the Rapid Preparation of Tissues for Classes in Histology, Prof. Simon H. Gage, Ithaca, N. Y.; (1) The Rotifera of Central Michigan, (2) Recent Methods of Investigating Microscopical Animals, Prof. D. S. Kellicott, Columbus, Ohio; Some Methods of Treating Nerve Tissue, Dr. William C. Krauss, Buffalo, N. Y.; (1) An Infallible Method of Preparing Injecting Gelatine and Injecting Small Animals, (2) Observations on Mounting, Dr. R. N. Reynolds, Detroit, Mich.; Résumé of the Past Year's Advance in Microscopy, Dr. Lee H. Smith, Buffalo, N. Y.; (1) A new Flash Light in Photography as Applied to Microscopy, (2) Postal Cards and Vegetable Fibers, (3) The Possibilities of the James Cement, with Many Fine Specimens, Dr. Thomas Taylor, Washington, D. C.; The Cancer Cell, Dr. L. Younghusband, Detroit, Mich.; Fresh Water Rhizopods of Oakland, Co., Stuart H. Perry, Pontiac, Mich.; Form and Endings of Striated Muscular Fibers, Mrs. S. S. Phelps Gage, Ithaca, N. Y.

The titles of Papers promised by other prominent workers have not yet been received. All who propose reading papers are requested to fill out the blanks enclosed and mail to the undersigned, who will forward them to Secretary Burrill. These suggestions apply also to the blanks for membership. A marked increase in membership is looked for at the Detroit meeting. Admission fee, $3.00; annual dues, $2.00, payable in advance.

Completion of manuscript prior to meeting will greatly aid the Publication Committee. Papers read before the Society may be published in any reputable journal, provided due acknowledgment is made that they are from the Proceedings of the American Society of Microscopists.

Negotiations relating to reduced railroad fares have been in progress. Should they be successful, due notice will be given.

The Local Committee at Detroit will issue circulars relating to the "Working Session," and the "Exhibition." They will supply badges and look after the general welfare of those attendant upon the Convention.

George E. Fell, M. D., *President*,
Buffalo, N. Y.

---

The American Public Health Association will hold its Eleventh Annual Meeting at Charleston, S. C., Tuesday, Wednesday, Thursday, and Friday, December 16, 17, 18, and 19, 1890.

---

The American Gynecological Society will hold its Fifteenth Annual Meeting in Buffalo, Tuesday, Wednesday and Thursday, September 16, 17, and 18, 1890.

CANADIAN MEDICAL ASSOCIATION, Montreal, June 25, 1890.—The twenty-third annual meeting of the Canadian Medical Association will be held in Toronto, on the 9th, 10th and 11th of September, next. Arrangements will be made with the Railroad and Steamboat Companies for a reduced traveling rate, and certificates entitling members to such reduction will be issued by the secretary on application. Members intending to present papers at this meeting, are requested to notify the secretary at as early a date as possible of title of the paper intended to be read.

JAMES BELL, M. D., *Secretary.*

### BOOKS AND PAMPHLETS RECEIVED.

Diseases of the Rectum and Anus. Their Pathology, Diagnosis, and Treatment. By Chas. B. Kelsey, A. B., M. D., Professor of Diseases of the Rectum, at the New York Post Graduate Medical School and Hospital; late Professor of Diseases of the Rectum at the University of Vermont, etc. Third edition, re written and enlarged, with two chromo-lithographs and 168 illustrations. Standard 8vo, pp. x.—483. New York: William Wood & Co. 1890.

Terminologia Medica Polyglotta. A Concise International Dictionary of Medical Terms. Compiled by Theodore Maxwell, M. D., Camb., B. Sc., Lond., F. R. C. S., Edin.; with the Assistance of Nine Collaborators. In Latin, English, French, German, Italian, and Spanish. 8vo, pp. xvi.—459. London: J. & A. Churchill. Paris: G. Masson. Philadelphia: P. Blakiston, Son & Co. 1890.

Annual of the Universal Medical Sciences. A Yearly Report of the Progress of the General Sanitary Sciences throughout the World. Edited by Charles E. Sajous, M. D., and Seventy Associate Editors. Five volumes. Philadelphia: F. A. Davis. 1890.

Familiar Forms of Nervous Disease. By M. Allen Starr, M. D., Ph. D., Professor of Diseases of the Mind and Nervous System, College of Physicans and Surgeons, New York. With illustrations, diagrams, and charts. 8vo, pp. xii.—339. New York: William Wood & Company. 1890.

Annual Report of the State Board of Charities. Fourth Year, 1889. Transmitted to the Legislature, January 15, 1890. 8vo, pp. 411. Albany: James B. Lyon, State Printer. 1890.

Practical, Sanitary, and Economic Cooking, adapted to persons of moderate and small means. By Mrs. Mary Hinman Abel. The Lomb Prize Essay. 12mo, pp. xii.—190. Published by The American Public Health Association. 1890.

Philosophy in Homeopathy. Addressed to the Medical Profession and to the General Reader. By Charles S. Mock, M. D., Professor of Materia Medica and Therapeutics in the Homeopathic College of the University of Michigan, at Ann Arbor. 12mo, pp. 174. Chicago: Gross & Delbridge. 1890.

Mineral Springs and Health Resorts of California, with a Clinical Analysis of every Important Mineral Water in the World. Illustrated. A Prize Essay. Annual Prize of the Medical Society of the State of California, awarded April 20, 1889. By Winslow Anderson, M. D., joint editor and publisher of the *Pacific Medical Journal*, Assistant Chair Medical Chemistry and Materia Medica, and Teacher of Chemistry in the Laboratories of the University of California, in the Medical and Dental Departments, etc. 8vo, pp. xxx.—384. San Francisco: The Bancroft Company. 1890. Price, $1.50. Special rates to Spring owners.

How to Examine for Life Insurance. By John M. Keating, M. D., President of the Association of Life Insurance Medical Directors, etc. 8vo, pp. viii.—204. Philadelphia: P. Blakiston, Son & Co. 1890.

Rheumatism and Gout. By F. LeRoy Satterlee, M. D., Ph. D., Professor of Chemistry and Therapeutics, in the New York College of Dentistry, etc. The Physicians' Leisure Library. Detroit: George S. Davis. 1890. Price, cloth, 50 cents; paper, 25 cents.

Wood's Medical and Surgical Monographs. Vol. VII., No. 1. July, 1890.

Eighteenth Annual Report of the Board of Inspectors of the House of Correction of the City of Chicago, and Reports of the Superintendent and City Physician to the Board, for the year 1889. Chicago: Knight & Leonard Co., Printers. 1890.

Menstruation and the Removal of Both Ovaries. By George J. Engelmann, A. M., M. D., St. Louis, Mo.: Reprint from the Transactions of the Southern Surgical and Gynecological Association, November, 1889.

The Professional Reference Lists. Designed for and intended as an advertising medium of the interests of Fred D. Van Horen, 265 Broadway, New York.

Illustrated Catalogue and Price List of Dr. Geo. H. Taylor's Remedial Apparatus. The Improved Movement Cure, 71 E. 59th street, New York.

Proceedings of the Philadelphia Obstetrical Society for February, March and April, 1890. Reprint from the *Annals of Gynecology and Pediatry.* Philadelphia: University of Pennsylvania Press. 1890.

Annual Report of the Department for the Insane of the Pennsylvania Hospital, for the year ending April 22, 1890. Presented to the 139th Annual Meeting of the Managers of the Pennsylvania Hospital. By John B. Chapin, M. D., Physician-in-Chief and Superintendent Philadelphia: Press of Ferris Brothers. 1890.

Climatology and Diseases of Southern California. By F. E. Bullard, A. M., M. D. Reprint from the *Southern California Practitioner.* Prize Essay of the California State Medical Society. 1890.

Another Hitherto Undescribed Disease of the Ovaries. Anomalous Menstrual Bodies. By Mary A. Dixon Jones, M. D. Brooklyn: Reprint from *The New York Medical Journal*, for May 10 and 17, 1890.

The Treatment of Local and General Peritonitis. By W. E. B. Davis, M. D., Birmingham, Ala. Reprint. Read before the Medical Association of the State of Alabama, April 13, 1890.

*The Buffalonian.* An Amateur Monthly Journal. McKibbin & Kingsley, Editors and Publishers. Vol. I. Nos. 1, 2 and 3. May, June, and July, 1890.

Supra-Vaginal Hysterectomy. Hysteromyomectomy with Suspension of the Stump in the Lower Angle of the Abdominal Incision. By Howard A. Kelly, M. D., Gynecologist to the Johns Hopkins Hospital, Baltimore. Reprint from the *Medical News*, June 28, 1890.

Extra-uterine Pregnancy. Papers read before the Obstetrical and Gynecological Society of Baltimore, Jan. 14 and Feb. 11, 1890. By Dr. G. W. Miltenberger, Dr. T. A. Ashby, and Dr. H. A. Kelly. Published by order of the Society.

Some Recent Cases in Pelvic and Abdominal Surgery. By L. S. McMurtry, M. D., Gynecologist to Saints Mary and Elizabeth Hospital, Louisville. Reprint *American Practitioner and News.* Read before the Kentucky State Medical Society, May 14, 1890.

The Use of Powdered Jequirity in Certain Affections of the Eye. By W. Cheatham, M. D., Lecturer on Diseases of the Eye, Ear, Throat and Nose, Medical Department, University of Louisville. Reprint *Journal American Medical Association*, June 28, 1890.

Recollections of General Grant, with an account of the presentations of the portraits of Generals Grant, Sherman, and Sheridan, at the U. S. Military Academy, West Point. By George W. Childs. Philadelphia: Collins' Printing House. 1890.

An Investigation into the Etiology of Phthisis. By Heneage Gibbes, M. D., Professor of Pathology, University of Michigan; and E. L. Shurly, M. D., Professor of Laryngology and Clinical Medicine, in the Detroit College of Medicine. Reprints from the *American Journal of the Medical Sciences*, May and July, 1890.

Abortion and its Effects. By Joseph Taber Johnson, A. M., M. D., Ph. D., Washington, D. C., Professor of Gynecology in the University of Georgetown, etc. Annual oration delivered April 23, 1890, before the Medical and Chirurgical Faculty of Maryland. Reprint from the *Maryland Medical Journal.*

Identité de la Dengue et de la Grippe-Influenza. Par le docteur Jules Rouvier, Professeur de Clinique Obstetricale et Gynécologique à la Faculté francaise de Médecine de Beyrouth, etc. Prix : un franc. Beyrouth (Syrie) Direction de la Revue Internationale de Bibliographic. 1890.

An Intra-Ligamentary Ovarian Cyst Successfully Treated by Iodine Injections. Reported by R. B. Rhett, Jr. Reprint.

De l'action Polaire Positive du Courant Galvanique Constant sur les microbes et En particulier sur la bactéridie Charbonneuse. Par M. M. Apostoli et Laquerrière. Paris. Fasciculus. Through Dr. H. E. Hayd.

Nine Months' Work in Abdominal Surgery. By Clinton Cushing, M. D., Professor of Gynecology, Cooper Medical College, San Francisco, Cal. Reprint *Pacific Medical Journal*, July, 1890.

Bureau of Education. Circulars of Information, Nos. 1, 2, and 3, 1890. Contributions to American Educational History. Edited by Herbert B. Adams. I. The History of Federal and State Aid to Higher Education in the United States, by Frank W. Blackmer, Ph. D. II. English-Eskimo and Eskimo-English Vocabularies. Compiled by Ensign Roger Wells, Jr., U. S. N., and Interpreter John W. Kelly. Washington: Government Printing Office. 1890.

The Therapeutical Applications of Peroxide of Hydrogen and Glycozone (Medical). By Charles Marchand, Chemist. Revised edition. New York. 1890.

HEALTH BULLETINS.

Tennessee State Board of Health, July 20, 1890.

Michigan State Board of Health, July 3, 1890.

New York State Board of Health, May, 1890.

Newport, R. I., Board of Health, June, 1890.

Abstract of Sanitary Reports. Vol. V., Nos. 27, 28, 29, 30. Marine Hospital Bureau. Washington, D. C.

Abstract of Proceedings of the Michigan State Board of Health Meeting, April 15, 1890.

MEDICAL COLLEGE ANNOUNCEMENTS FOR 1890–91.

Albany Medical College and catalogue.

Baltimore Medical College and catalogue.

Bellevue Hospital Medical College, with list of Graduates for 1890.

College of Physicians and Surgeons of Chicago.

College of Physicians and Surgeons in the City of New York, with catalogue.

Long Island College Hospital, Brooklyn, with catalogue.

McGill University. Annual Calendar. Faculty of Medicine.

Meharry Medical Department, Central Tennessee College, Nashville.

National Medical College, Medical Department, Columbia University, Washington, D. C.

New York Medical College and Hospital for Women.

New York Polyclinic and Hospital.

Niagara University, Medical Department.

University of the City of New York, Medical Department.

Western Pennsylvania Medical College, Pittsburg.

